# Supplementary material for: Substrates bind to residues lining the ring of asymmetrically engaged bacterial proteasome activator Bpa
Source: Nat Commun. 2025 Mar 28;16:3042. doi: 10.1038/s41467-025-58073-1 (PMC11953334; doi:10.1038/s41467-025-58073-1)
Supplement: Supplementary file 1 — Supplementary Information [file 41467_2025_58073_MOESM1_ESM.pdf]

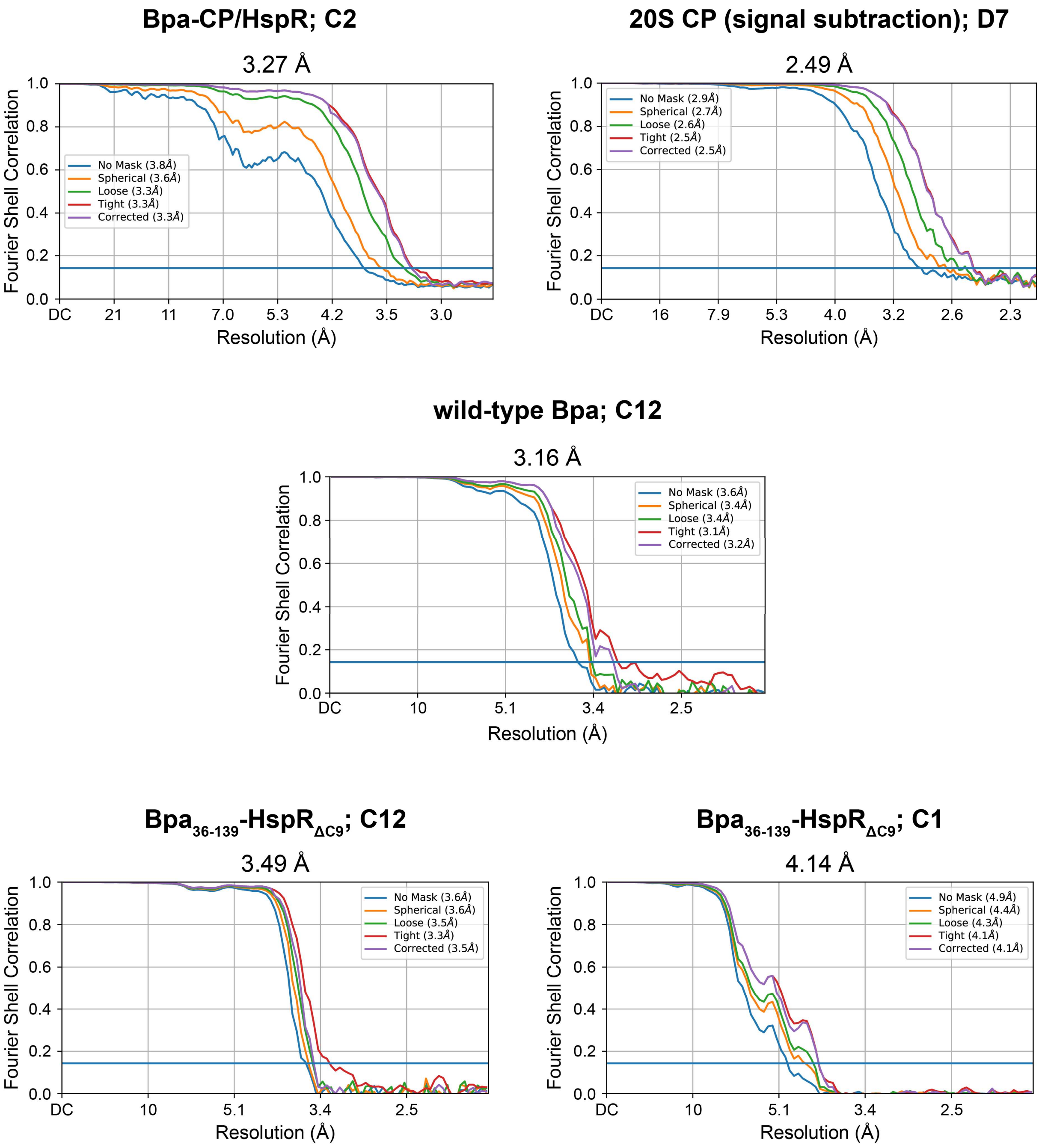

Supplementary Fig. 1. Fourier shell correlation (FSC) curves of the cryo-EM maps presented in this study. The plots were generated in cryoSPARC.

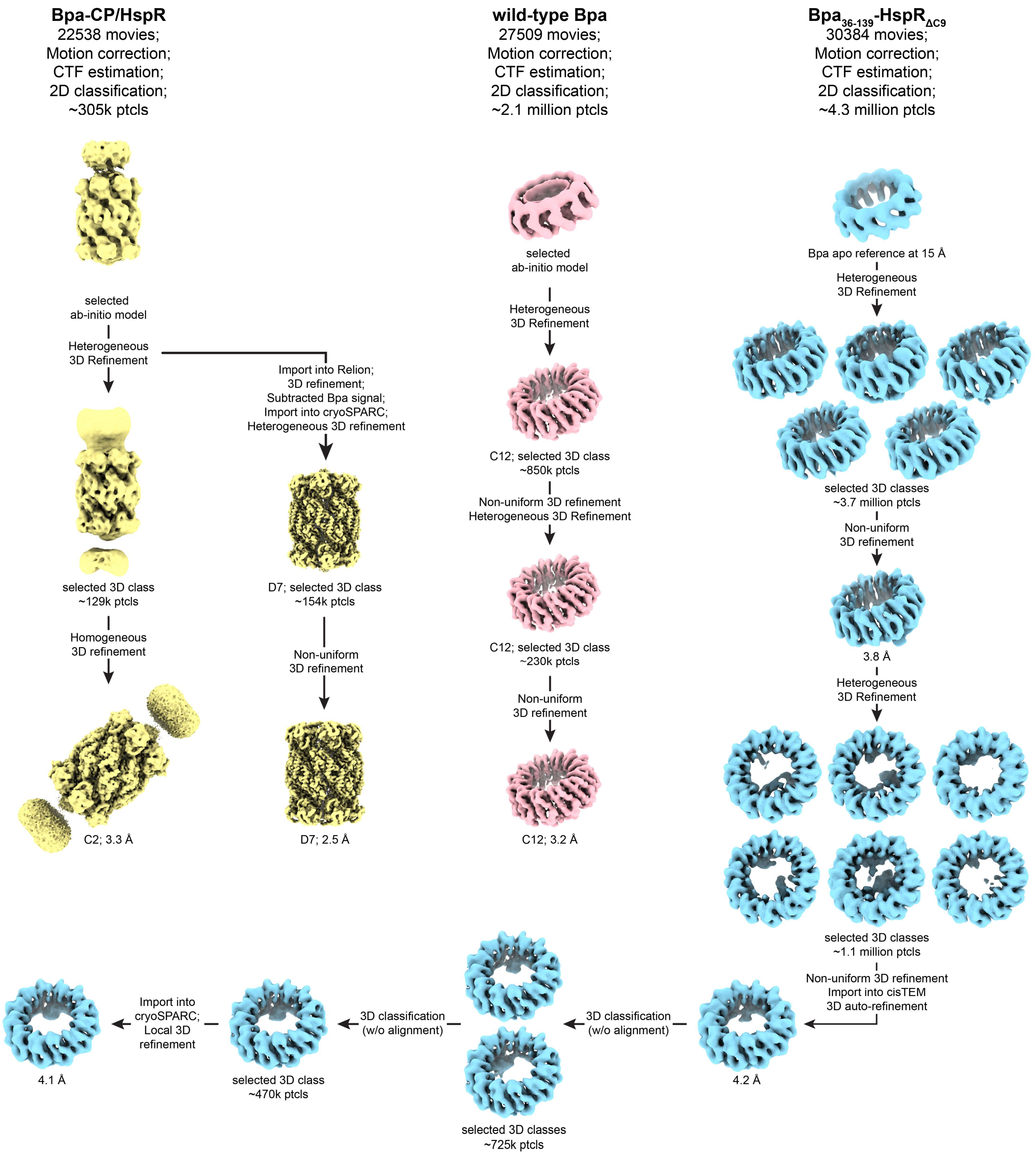

Supplementary Fig. 2. Schematic representation of the cryo-EM single-particle data processing. See Materials and Methods for detailed description.

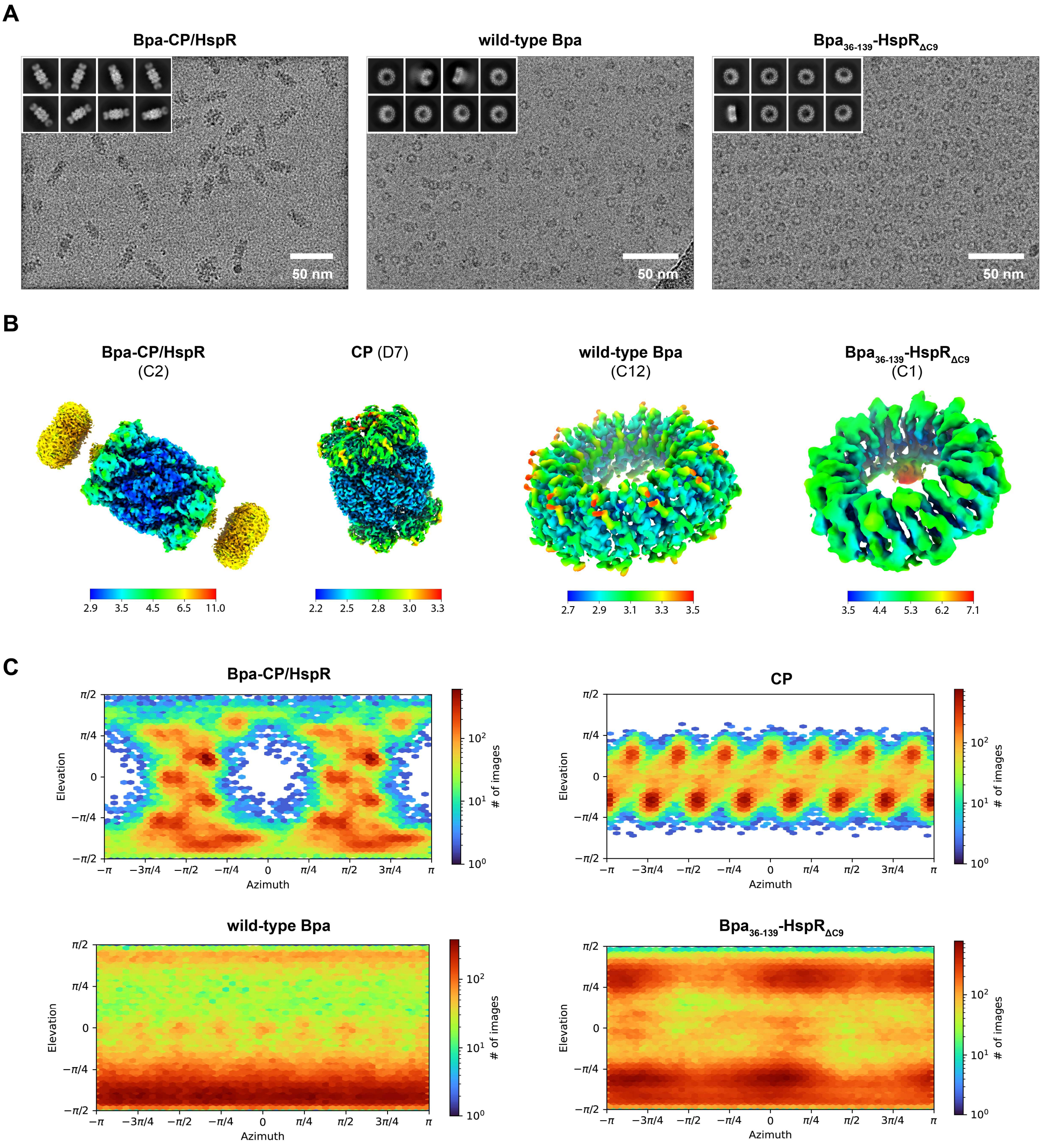

Supplementary Fig. 3. Additional cryo-EM processing data. A) Representative micrographs of the corresponding samples with examples of 2D class-averages shown as insets. B) Cryo-EM maps colored by local resolution. The overfitted Bpa region in the Bpa-CP/HspR map was resolved to low resolution. C) Angular distribution of particles used in the final 3D reconstructions.

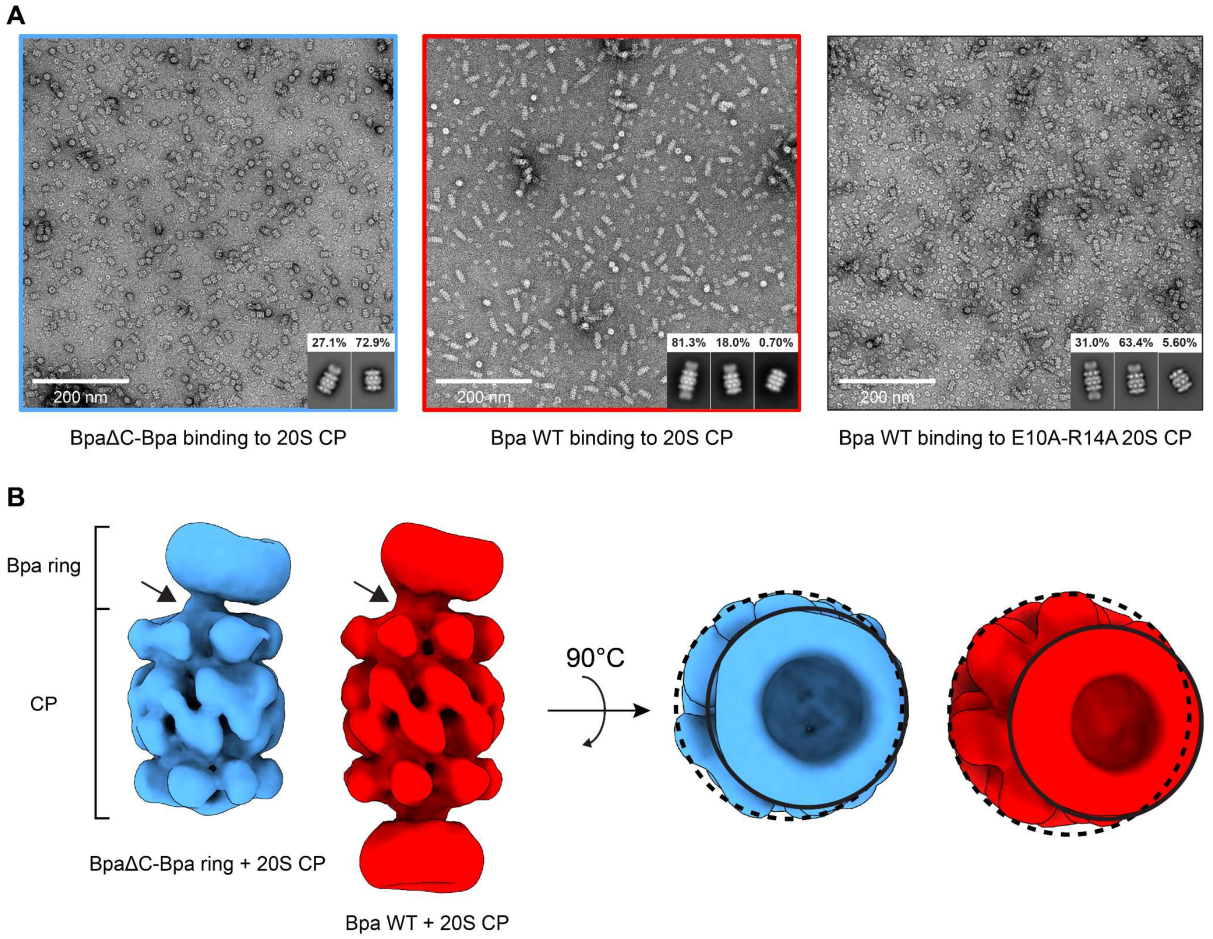

Supplementary Fig. 4. Negative stain EM analysis of Bpa-CP complexes. A) Representative micrographs of the corresponding samples with examples of 2D class-averages shown as insets. Complexes used: the mixed Bpa oligomer (BpaΔC-Bpa) with CP (left), wild-type Bpa with CP (middle), wild-type Bpa with helix H0-mutated CP (right). B) Side and top views of the negative stain EM maps obtained for BpaΔC-Bpa and wild-type Bpa in complex with CP. Density differences between the Bpa rings and the CP are indicated by a black arrow.

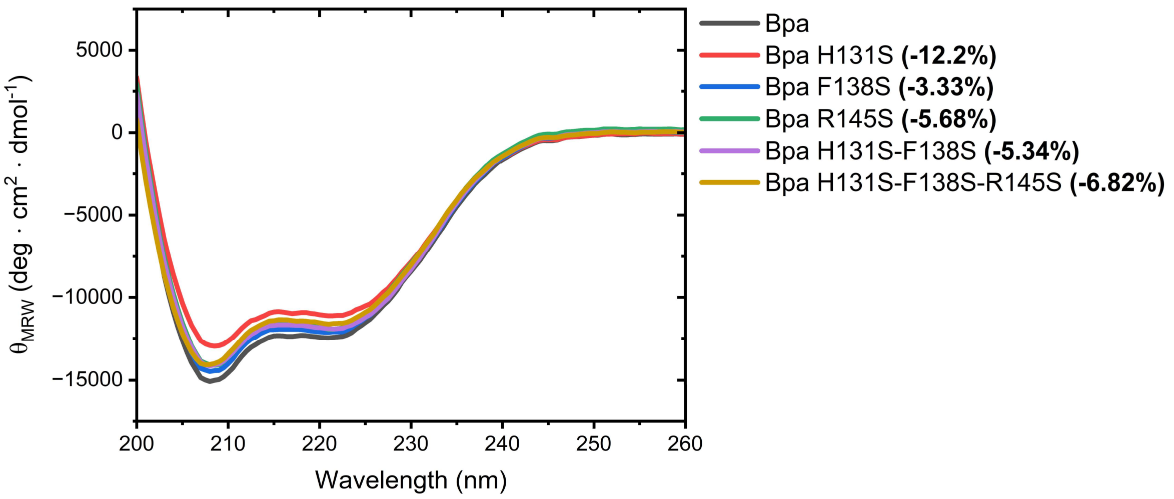

Supplementary Fig. 5. CD spectra of Bpa variants. CD spectra of wild-type Bpa, Bpa H131S, F138S, R145S single point mutants, Bpa H131S-F138 double point mutant, Bpa H131S-F138-R145 triple point mutant. All CD spectra were measured from 260 nm to 200 nm and are an average of three replicates with five acquisitions each. Signals were buffer-corrected and normalized to protein amount.

Supplementary Table 1. Cryo-EM data collection and refinement statistics.

	Bpa-CP/HspR		wild-type Bpa	Bpa_{36-139}–$HspR_{\Delta C9}$	
EMDB code	EMD-19150<br>EMD-19151 (CP)		EMD-19162	EMD-19159	EMD-19153
PDB code			8RGX		
**Data collection and processing**					
Magnification	x130000		x165000	x165000	
Camera	K3		K3	K3	
Voltage (kV)	300		300	300	
Electron exposure (e^-/Å^2)	80		80	80	
Defocus range (μm)	1.2–2.6		1.2–2.6	1.2–2.6	
Pixel size (Å/pixel)	0.66		0.51	0.51	
	**Bpa-CP**	**CP**			
Symmetry imposed	C2	D7	C12	C12	C1
Initial particle images (no.)	305468	305468	2108536	4330895	4330895
Final particle images (no.)	128712	154384	229813	414680	469948
Map resolution (Å)	3.3	2.5	3.2	3.5	4.1
FSC threshold	0.143	0.143	0.143	0.143	0.143
Map resolution range (Å)	2.9-11	2.3-3.3	2.7-3.5	2.8-3.3	3.5-7.1
**Refinement**					
	**CP**				**C1**
Initial model used (PDB)	5LZP		5LFJ		Bpa (this study)
Model resolution (Å)	2.6		3.2		4
FSC threshold	0.5		0.5		0.5
Nonhydrogen atoms	46802		9924		9024
Protein residues	6216		1260		1140
B-factors (Å^2)					
Protein	0.0/91.7/25.6		41.2/102.9/53.3		77.5/143.5/90.5
R.m.s. deviations					
Bond length (Å)	0.003		0.004		0.003
Bond angles (°)	0.529		0.479		0.485
Ramachandran statistics					
Favored (%)	97.94		99.03		99.91
Allowed (%)	2.06		0.97		0.09
Outliers (%)	0		0		0
MolProbity score	1.13		1.24		1.52
Clash score	3.26		4.67		7.23
Poor rotamers (%)	0.3		0.76		1.46

Crosslinked peptides	Protein a	Protein b	Bpa res. #	HspR res. #
ZGXSLTDLVEQPAK-ZAKNPK-a1-b3	Bpa	HspR	N-term (36)	N-term (1)
ZGXSLTDLVEQPAK-NPKDGESR-a1-b3	Bpa	HspR	N-term (36)	5
ZGMSLTDLVEQPAK-NPKDGESR-a1-b3	Bpa	HspR	N-term (36)	5
IGTMIKQLLEEVR-NPKDGESR-a6-b3	Bpa	HspR	55	5
ZGXSLTDLVEQPAK-REVAVVPKST-a1-b8	Bpa	HspR	N-term (36)	114
ZGXSLTDLVEQPAK-EVAVVPKST-a1-b7	Bpa	HspR	N-term (36)	114
ZGMSLTDLVEQPAK-EVAVVPKST-a1-b7	Bpa	HspR	N-term (36)	114
ZGMSLTDLVEQPAK-ZAKNPK-a1-b3	Bpa	HspR	N-term (36)	N-term (1)
IGTXIKQLLEEVR-NPKDGESR-a6-b3	Bpa	HspR	55	5
ZGMSLTDLVEQPAK-REVAVVPKST-a1-b8	Bpa	HspR	N-term (36)	114
IGTMIKQLLEEVR-REVAVVPKST-a6-b8	Bpa	HspR	55	114
IGTMIKQLLEEVR-EVAVVPKST-a6-b7	Bpa	HspR	55	114
ZGMSLTDLVEQPAK-ZAKNPKDGESR-a1-b6	Bpa	HspR	N-term (36)	N-term (1)
IGTXIKQLLEEVR-EVAVVPKST-a6-b7	Bpa	HspR	55	114
IGTXIKQLLEEVR-REVAVVPKST-a6-b8	Bpa	HspR	55	114
ZGXSLTDLVEQPAK-ZAKNPKDGESR-a1-b6	Bpa	HspR	N-term (36)	N-term (1)

Supplementary Table 2. List of crosslinked peptide pairs representing protein-protein contacts identified from the reaction of Bpa_{36-139} and $HspR_{\Delta C9}$ with BS^3. "Z" represents the reaction with the N-terminus of the respective protein. "X" denotes an oxidized methionine residue.
